# Sampling of *Culicoides* with nontraditional methods provides unusual species composition and new records for southern Spain

**DOI:** 10.1186/s13071-024-06414-2

**Published:** 2024-08-12

**Authors:** Mikel Alexander González, Sergio Magallanes, Daniel Bravo-Barriga, Victor Sarto i Monteys, Josué Martínez-de la Puente, Jordi Figuerola

**Affiliations:** 1 Estación Biológica de Doñana (EBD, CSIC), Seville, Spain; 2 Ciber de Epidemiología y Salud Pública (CIBERESP), Madrid, Spain; 3 Departamento de Salud Animal, Grupo de Investigación en Salud Animal y Zoonosis (GISAZ), Facultad de Veterinaria, Universidad de Córdoba, Córdoba, Spain; 4 Institut de Ciència i Tecnologia Ambientals (ICTA), Entomology, Plants and Health, Universitat Autònoma de Barcelona, Bellaterra, Spain

**Keywords:** Biting midges, Bluetongue vectors, Medical entomology, Natural habitats, Trapping methods, Species composition

## Abstract

**Background:**

*Culicoides* midges have been well-studied in Spain, particularly over the last 20 years, mainly because of their role as vectors of arboviral diseases that affect livestock. Most studies on *Culicoides* are conducted using suction light traps in farmed environments, but studies employing alternative trapping techniques or focusing on natural habitats are scarce.

**Methods:**

In the present study, we analyze *Culicoides* captured in 2023 at 476 sites in western Andalusia (southern Spain) using carbon dioxide-baited Biogents (BG)-sentinel traps across different ecosystems.

**Results:**

We collected 3,084 *Culicoides* midges (3060 females and 24 males) belonging to 23 species, including the new species *Culicoides grandifovea* sp. nov. and the first record of *Culicoides pseudolangeroni* for Europe. Both species were described with morphological and molecular methods and detailed data on spatial distribution was also recorded. The new species showed close phylogenetic relations with sequences from an unidentified *Culicoides* from Morocco (92.6% similarity) and with *Culicoides kurensis*. *Culicoides imicola* was the most abundant species (17.4%), followed by *Culicoides grandifovea* sp. nov. (14.6%) and *Culicoides kurensis* (11.9%). Interestingly, *Culicoides montanus* was the only species of the *obsoletus *and *pulicaris* species complexes captured, representing the first record of this species in southern Spain. A total of 53 valid *Culicoides* species have been reported in the area, with 48 already reported in literature records and 5 more added in the present study. Information on the flight period for the most common *Culicoides* species is also provided.

**Conclusions:**

To the best of our knowledge, our study represents the most comprehensive effort ever done on nonfarmland habitats using carbon-dioxide baited suction traps for collecting *Culicoides*. Our data suggests that using carbon dioxide traps offers a completely different perspective on *Culicoides* communities compared with routinely used light traps, including the discovery of previously unrecorded species.

**Graphical Abstract:**

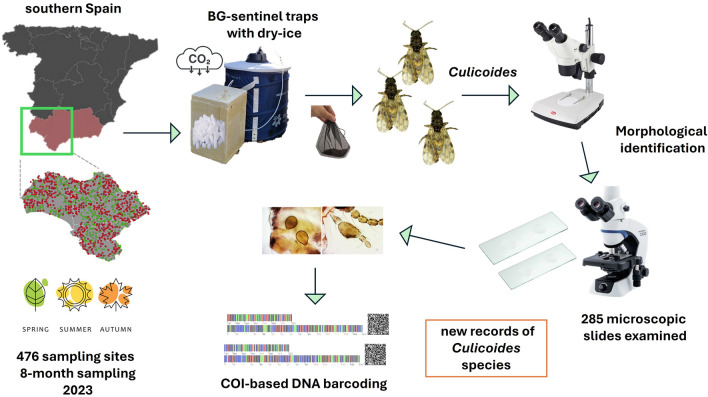

**Supplementary Information:**

The online version contains supplementary material available at 10.1186/s13071-024-06414-2.

## Background

*Culicoides* is a taxonomically diverse genus of tiny hematophagous insects belonging to the family Ceratopogonidae. The number of *Culicoides* species has increased over the last few years in Europe, particularly those belonging to the subgenus *Culicoides,* due to the rise of molecular approaches [1–5]. *Culicoides* are vectors of arboviruses of the *Orbivirus* genus, such as the African horse sickness virus (AHSV), bluetongue virus (BTV), and Schmallenberg virus (SV) [6–9]. The recent outbreaks of epizootic hemorrhagic disease (EHD) in Spain, a virus transmitted by *Culicoides*, which primarily affects cervids and livestock [10, 11], has renewed the interest in this group. Furthermore, *Culicoides* are also vectors of parasites infecting nonmammal hosts, including the avian malaria-like parasites of the genus *Haemoproteus* [12].

Due to their minute size, the identification of *Culicoides* species across Europe has remained challenging. Morphological diagnostic characters that are commonly used for identification are often difficult to observe. Wing spot patterns are of primary importance in species diagnosis [13–15]. However, some *Culicoides* species bear faint spots or lack a defined wing pattern resulting in clear wings without markings, these being routinely grouped as “other *Culicoides* species” in large faunistic studies [16, 17]. Considering that about 25% of the European *Culicoides* are faint or unspotted species, the study of *Culicoides* communities requires mounting specimens in slides. This task requires skills and is laborious, time-consuming, and impracticable when a large number needs to be identified and may be incompatible with the preservation of specimens for pathogen surveillance. Many studies published in Europe only focused on species with wing patterns, which usually correspond to vectors involved in epizootics (subgenus *Culicoides* and *Avaritia*) [18–20]. However, the identification of nontarget *Culicoides* fauna should also be undertaken, not only to improve the faunistic inventories, but also for a better characterization of other unknown potential vectors that might arise in future epizootics [21].

Suction light traps, particularly the commercially available Onderstepoort Veterinary Institute (OVI) trap and the ultraviolet (UV)–Center for Disease Control (CDC) downdraft suction trap are the most commonly used traps for the collection of *Culicoides* [22]. These traps are routinely chosen for their ease of installation, provision of standardized data among studies, and ability to collect a reasonable numbers of vector species when present [22]. However, as shown in other insect groups, using alternative approaches to sample *Culicoides* may provide new opportunities to collect species attracted to other stimuli [22, 23].

Since information on the composition and distribution of *Culicoides* species is a prerequisite to understand the epidemiology of *Culicoides*-borne pathogens, surveillance contributes to the development of effective strategies for disease prevention and control. In Spain, until 2012, 81 *Culicoides* species were recorded [24], and in the subsequent 12 years, to our knowledge, 5 more species were added [2, 25–27]. However, current information of the *Culicoides* fauna differs between regions, with southern Spain being comparatively understudied in spite that AHSV [28, 29], BTV [30] and EHD [11] outbreaks occurred in the area in 1956–1960 and 2004–2024. This area has been severely affected by West Nile virus outbreaks in recent years [31], and a large effort is being done for the characterization of mosquito communities across the territory. In addition to mosquitoes, *Culicoides* biting midges are often captured in these traps. Here, we conducted an extensive monitoring of *Culicoides* in several diverse environments using an alternative sampling method to improve the knowledge of the *Culicoides* distribution in the area. In addition, we carried out a bibliographic review of the *Culicoides* species recorded in southern Spain.

## Methods

### Study area, design, and trapping

**Fig. 1:**
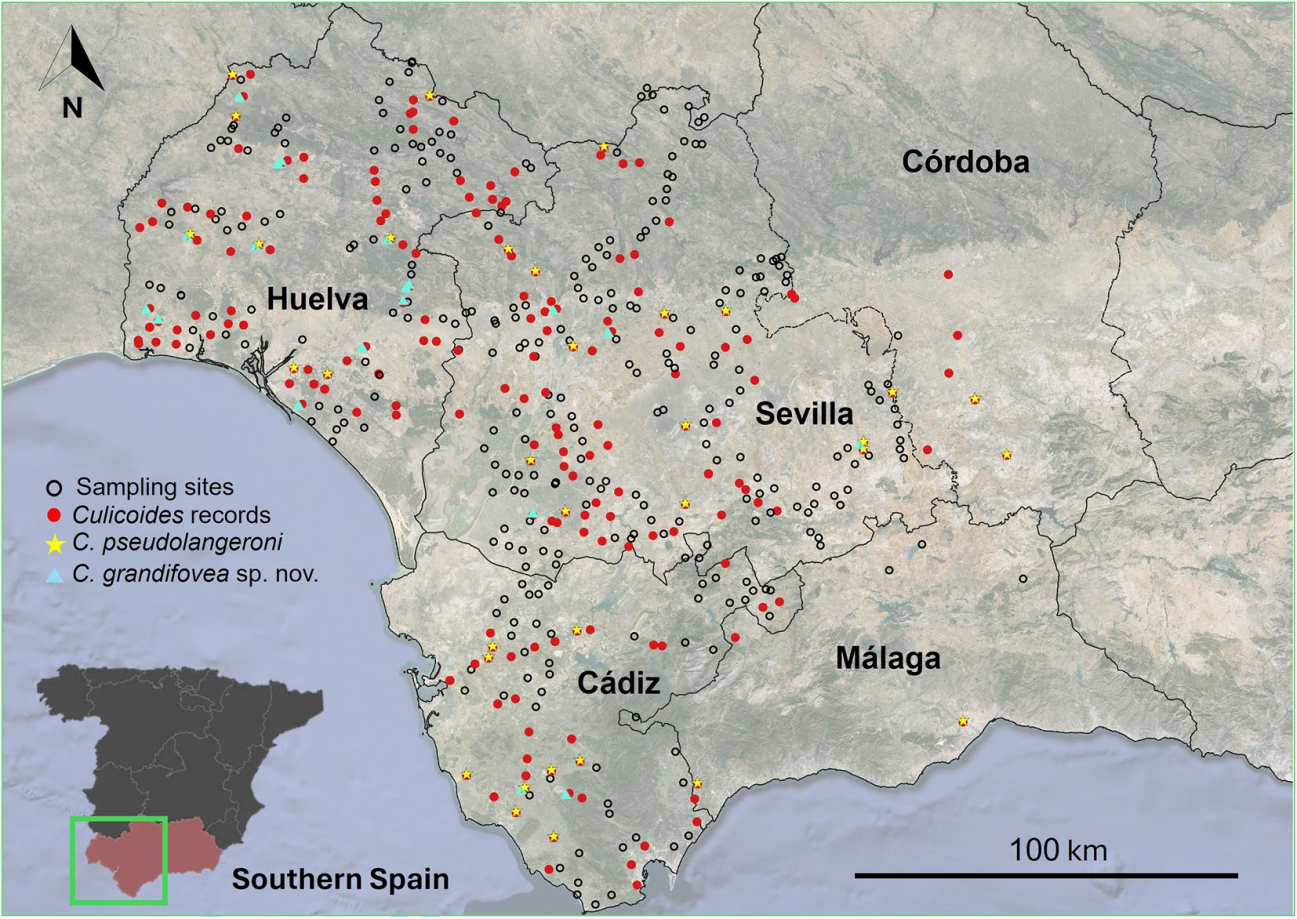
Map of the 476 sampling sites in the five western provinces of Andalusia (southern Spain). Empty circles: negative sampling sites for the presence of *Culicoides*. Red circles: sampling sites with captures of *Culicoides*. Yellow starts: sampling sites with captures of *Culicoides pseudolangeroni*. Blue triangles: sampling sites with captures of *Culicoides grandifovea* sp. nov. The map was created using QGIS software (QGIS version 3.32)

The study was conducted in the provinces of Huelva, Sevilla, Málaga, Córdoba, and Cádiz of the Andalusia region (southern Spain) (Fig. 1). This region is characterized by a Mediterranean climate with mild winters with irregular precipitations and dry, hot, and sunny summers. Some areas experience the hottest temperatures in the country during summer (> 45 °C). In 2023, when this study was conducted, the average year-round temperature was approximately 19 °C, with more than 290 days of sunshine. January was the coldest month and August the hottest.

*Culicoides* biting midges were collected using two different surveys. In the first survey (first study hereinafter), a total of 450 sampling sites were sampled three times (spring, summer, and autumn) from April to November 2023. The total area sampled covered 31,500 km^2^ across the provinces of Sevilla, Cádiz, and Huelva, resulting in an overall trapping effort of approximately 1350 trapping days (Fig. 1). To ensure systematic coverage, the area was divided into grids (5 km × 5 km) with one sampling point selected in each grid. The sampling points and routes were determined at the beginning of the study to design fieldwork tracks that would maximize the number of traps surveyed each day. The order of sampling of these predefined tracks was randomly established to prevent associations between time and land-use geography. The second survey (second study) corresponds to the Junta de Andalusia mosquito survey program, with 26 sampling sites: eight in Sevilla and eight in Cádiz sampled at weekly intervals and six in Córdoba and four in Málaga sampled every 2 weeks, in both cases from 15 June to 23 November 2023 (i.e., 436 trapping days) (Fig. 1).

In all cases, insects were sampled using the commercially available BG-Sentinel model 2 trap (Biogents, Regensburg, Germany). These traps were baited with approximately 1.2 kg of dry ice in a polystyrene box to generate a continuous flow of carbon dioxide at the entrance of the trap, placed on the ground and were operated for 24 h. Traps were set in shaded and sheltered areas to avoid direct sunlight and wind, to increase captures and reduced the risk of vandalism. Samples were preserved in dry ice while transported to the laboratory and subsequently stored at −80 °C.

### Morphological identification and molecular analysis

Frozen insects were immediately separated into groups on a chill table (BioQuip, Rancho Dominguez, CA) under the stereomicroscope. *Culicoides* midges were sorted out by sex and feeding status, and then were separated into species with distinctive wing patterns (i.e., *Culicoides jamaicensis*, *Culicoides newsteadi*, *Culicoides imicola*, *Culicoides circumscriptus*, etc.) and those with plain wings. Specimens with unspotted wings and/or unknown wing-patterned species were further examined on the basis of other traits such as body size, color, thorax pattern, wing features, and palpi. A subset of 285 distinctive *Culicoides* specimens was then individually dissected into different body parts (head, thorax, wings, and abdomen), mounted with Hoyer's medium on glass slides using needles (0.5 mm diameter) and dried at room temperature for 7 days. Key diagnostic structures of specimens were examined using a composed optical microscopy, employing a combination of three identification keys [13, 14, 32].

Length of every palpus and flagellar segments, wing length (from basal arculus to wing tip) and wing width (from R_2_ to vein Cu_1_), and spermathecae were photographed and measured under the optical microscope (Zeiss, Axioscope, UK) with a digital camera (Axioram 208 model) for the unsubscribed species and the new record *Culicoides* species (to complete the original descriptions). Measurements of the different parts of the specimens were performed using the Zeiss analyzing software. The area of the sensorial pit in the third palpus was also calculated with the “area calculator tool” in the new *Culicoides* species identified in this study. Antennal ratio (AR) was calculated as XI–XV antennal segments divided with segments III–X, and the Palpal ratio (PR) was calculated as length of segment III divided with the greatest breadth of the segment III.

The barcoding region of specimens of *C. pseudolangeroni* (*n* = 5) and *C. grandifovea* sp. nov. (*n *= 6) together with other plain-winged sibling species (specifically *Culicoides indistinctus* and *Culicoides kurensis*) (*n* = 4) were molecularly characterized. The head of each specimen was slide-mounted as previously described whereas the rest of the body was used for molecular analyses. Genomic DNA was extracted from each sample using the Maxwell^®^16 LEV Blood and Tissue DNA kit following the manufacturer's protocol. A 658 bp fragment of the cytochrome Oxidase Subunit I (COI) gene was amplified and sequenced following Folmer et al. [33]. The presence of amplicons was verified on 1.5% agarose gels. The amplified products were sequenced on both strands using Capillary Electrophoresis Sequencing by UCM (Madrid, Spain), and a consensus sequence was generated using Geneious v.2020.0.3 [34]. Species-level identity was determined with a threshold of > 99% identity score using BLASTn (https://blast.ncbi.nlm.nih.gov/Blast.cgi). Nucleotide sequences generated during this study were deposited in the DNA Data Bank of Japan (DDBJ: https://www.ddbj.nig.ac.jp/index-e.html).

### Phylogenetic analyses

A total of 24 *Culicoides* species (55 sequences) were included in the phylogenetic analysis. A sequence of *Atrichopogon levis* (GU804122) was used as outgroup. Phylogenetic reconstructions were conducted using the maximum likelihood optimization criterion, employing the GTR + F + I + G4 model as defined by IQ-TREE [35] and model selection was based on Akaike information criterion. The robustness of the resulting ML trees was evaluated using SH-aLRT (Shimodaira–Hasegawa-like approximate likelihood ratio test) and 1000 bootstraps. The tree was visualized using FigTree v1.4.2 (http://tree.bio.ed.ac.uk/software/figtree/). Finally, neighbor–net networks (NNn) were constructed using distance matrices corrected with the Kimura two-parameter model [36].

### Literature review

A checklist of *Culicoides* species reported in southern Spain was compiled through a systematic review following the guidelines outlined by Haddaway et al. [37]. The bibliographic investigation involved using various databases of scientific and public journals such Web of Science, Google Scholar, Scopus, Dialnet, PubMed, Redalyc, SciELO, BioOne, ScienceDirect, ResearchGate, and REDIB to identify articles released from 1900 to 2023. Our research included keywords in English and Spanish of the following combination of terms in the title, abstract and keywords: “*Culicoides*” OR “jejenes” OR “Ceratopogonidae” AND “Spain” as well as variations combining these keywords with specific regions such as “Andalucía,” “Andalusia,” “Iberia,” “Iberian,” and “Peninsula.” Combinations such as “*Culicoides*” AND “South Spain” and similar pairs with “jejenes” and “Ceratopogonidae” were also used. Additional articles were obtained from references from the articles reviewed. Duplicated publications and works that do not explicitly mention the *Culicoides* species or place of capture, were removed from the review. A total of 24 publications were included in the present study.

## Results

At least 48 species of *Culicoides* had been cited in Andalusia in studies published between 1900 and 2023 (Table 1). In our study, we collected 3,084 *Culicoides* specimens (3060 females and 24 males) in both sampling surveys (*n* = 2644 in the first one and 440 in the second one), representing 23 valid *Culicoides* species, four *Culicoides* variations and five undetermined taxa (Table 2). Our sampling includes, at least, five new records (Table 1–2) for Andalusia, including *Culicoides montanus* within the Obsoletus group, therefore totalizing 53 valid species for this region. This study includes a new species hereinafter referred to as *C. grandifovea* sp*.* nov. and a new record for Europe (*C. pseudolangeroni*). *Culicoides jumineri* near *bahrainensis* was also the first record for Spain, but it is not included as valid species. With these two new records, 88 *Culicoides* species have been reported in Spain, with about 60% of them present in Andalusia. Our study accounted for 19, 16, 17, 7, and 2 *Culicoides* species collected in the provinces of Huelva, Cádiz, Sevilla, Córdoba, and Málaga, respectively (Table 1).

**Table 1:** Updated checklist of *Culicoides* species reported in southern Spain based on literature records (1900–2023) and the present study (2023)

	*Culicoides* species	Literature records											Present study				
		Reference	Collection method	HU	CA	SE	CO	MA	JA	AL	GR		HU	CA	SE	CO	MA
1	*C. cataneii* Clastrier, 1957	^3–5,7^	SLT		X		X	X					X	X	X		
2	*C. cryptipulicaris* sp. nov. 2017^a^	^20^	SLT					X									
3	*C. bahrainensis* Boorman, 1989^b^	^5,7^	SLT	NA													
4	*C. begueti* Clastrier, 1957	^4^	SLT	X	X	X		X						X	X	X	
5	*C. brunnicans* Edwards, 1939	^2^	SLT				X										
6	*C. circumscriptus* Kieffer, 1918	^2–5,7 11,13,14,17,21,24^	SLT + BN	X	X	X	X	X	X	X	X		X	X	X	X	X
7	*C. chiopterus* (Meigen, 1830)	^4,7^	SLT		X			X									
8	*C. corsicus* Kremer, LeBerre & Beaucournu- Saguez, 1971	^4^	SLT	X		X				X			X	X	X		
9	*C. dewulfi* Goetghebuer, 1936	^16^	SLT	NA													
10	*C. duddingstoni* Kettle & Lawson, 1955	^2^	SLT				X										
11	*C. fagineus* Edwards, 1939	^2,5,7^	SLT				X							X	X		
12	*C. fascipennis* (Staeger, 1839)	^3,13^	SLT				X										
13	*C. festivipennis* Kieffer, 1914	^2–5,7,13,24^	SLT + BN	X	X	X	X	X			X		X		X		
14	*C. gejgelensis* Dzhafarov, 1964	^2,5,7^	SLT				X						X				
15	*C. griseidorsum* Kieffer, 1818	^2^	SLT				X										
16	***C. grandifovea*** sp. nov.		SLT										X	X	X		
17	***C. haranti*** Rioux, Descous & Pech, 1959		SLT										X		X		
18	*C. helveticus* Callot, Kremer & Deduit, 1962	^4^	SLT		X				X				X				
19	*C. heteroclitus* Kremer & Callot, 1965	^3,13^	SLT				X										
20	***C. indistinctus*** Khalaf, 1961		SLT										X	X	X		
21	*C. imicola* Kieffer, 1913	^2–11, 13,15–21,23^	SLT	X	X	X	X	X	X	X	X		X	X	X	X	
22	*C. jamaicensis* Edwards, 1922^c^	^12,21^	SLT					X		X			X	X	X	X	
23	*C. jumineri* Callot & Kremer, 1969	^3^	SLT				X										
24	*C. kibunensis* Tokunaga, 1937	^4,24^	SLT + BN			X	X				X						
25	*C. kurensis* Dzhafarov, 1960	^2,5,7^	SLT				X						X	X	X		
26	*C. longipennis* Khalaf, 1957	^3–5,7^	SLT		X		X	X					X		X		
27	*C. marcleti* Callot, Kremer & Basset, 1968	^5,7^	SLT				X										
28	*C. maritimus* Kieffer, 1924	^3,4,13,24^	SLT	X	X	X	X	X									
29	***C. minutissimus*** (Zetterstedt, 1855)												X	X			
30	***C. montanus*** Shakirzjanova, 1962												X				
31	*C. newsteadi* Austen, 1921^d^	^3–5,7,10,13,15^	SLT	X	X	X	X	X	X	X	X		X	X	X	X	
32	*C. nubeculosus* (Meigen, 1830)	^13, 16^	SLT	NA										X	X		
33	*C. obsoletus* (Meigen, 1818)	^4–5,7, 13, 15–17^	SLT	X	X	X	X	X	X	X	X						
34	*C. odiatus* Austen, 1921	^2,5,7,10^	SLT				X										
35	*C. parroti* Kieffer, 1922	^2,5,7,13^	SLT	X	X	X	X	X									
36	*C. pictipennis* (Staeger, 1839)	^4,13,24^	SLT	X	X			X									
37	*C. poperinghensis* Goetghebuer, 1953	^4^	SLT		X												
38	***C. pseudolangeroni*** Kremer, Chaker and Delecolle, 1981												X	X	X	X	X
39	*C. pulicaris* (Linnaeus, 1758)^e^	^4–5,7,11–17,19^	SLT	X	X	X	X	X	X	X	X						
40	*C. punctatus* (Meigen, 1804)	^5,7,13,15^	SLT	X	X	X	X	X	X	X							
41	*C. puncticollis* (Becker, 1903)	^1–3,7,13^	SLT	X	X	X	X	X									
42	*C. quasipulicaris* sp nov. 2017^a^	^20^	SLT		X												
43	*C. reconditus* Campbell and Pelham-Clinton, 1960	^24^	BN								X						
44	*C. riethi* Kieffer, 1914	^4–5,7^	SLT	X	X	X	X	X									
45	*C. saevus* Kieffer, 1922	^2^	SLT				X							X			
46	*C. sahariensi*s Kieffer, 1923	^4–5,7^	SLT				X						X	X	X	X	
47	*C. segnis* Campbell & Clinton, 1959	^4^	SLT	X	X			X		X							
48	*C. scoticus* Downes & Kettle, 1952	^7,16^	SLT				X										
49	*C. shaklawensi*s Khalaf, 1957	^3–4,7^	SLT		X		X	X					X				
50	*C. tauricus* Gutsevich, 1959	^22^	SLT								X						
51	*C. truncorum* Edwards, 1939	^24^	BN								X						
52	*C. univittatus* Vimmer, 1932	^4,5,7,10,13^	SLT	X		X				X							
53	*C. vidourlensis* Callot, Kremer, Molet & Bach, 1968	^2^	SLT				X										

New *Culicoides* records for Andalusia in the present study are in bold

^1^ [39], ^2^ [40], ^3^ [41], ^4^ [42], ^5^ [43], ^6^ [44], ^7^ [45], ^8^ [46], ^9^ [28], ^10^ [47], ^11^ [48], ^12^ [49], ^13^ [50], ^14^ [51], ^15^ [52], ^16^ [53],^17^ [54], ^18^ [55], ^19^ [56], ^20^ [2], ^21^ [25], ^22^ [57], ^23^ [19],^24^ [58]

*HU* Huelva, *CA* Cádiz, *SE* Sevilla, *CO* Córdoba, *MA* Málaga, *JA* Jaén, *GR* Granada, *AL* Almería, *SLT* suction light traps, *BN* bird nest, *NA* no information on the province is provided

^a^Species recently described by both molecular barcoding and morphological approaches[2]

^b^The taxonomic status of this species is not fully studied, and various synonyms have been proposed

^c^This species previously known as *C. paolae* has been proposed to be the same species than the American *C. jamaicensis*[38]

^d^This species within the Pulicaris group have been routinely gathered as *C. pulicaris*; thus, the distribution status is incomplete

^e^Previous studies based on DNA barcoding have proposed the existence of other phenotypes and genotypes[3]

**Table 2:** Number of *Culicoides* biting midges collected in 476 sampling sites (first study plus second study) in Andalusia (southern Spain) by using BG traps baited with carbon dioxide during 2023

*Culicoides* species	First study (%)	Second study (%)	Total (%)
*C. imicola*	453 (17.1)	85 (19.3)	538 (17.4)
*C. grandifovea* sp. nov. ^*1*^	449 (17.0)	0 (0.0)	449 (14.6)
*C. kurensis*	354 (13.4)	14 (3.2)	368 (11.9)
*C. pseudolangeroni *^*2*^	251 (9.5)	112 (25.5)	363 (11.8)
*C. circumscriptus*	285 (10.8)	50 (11.4)	335 (10.9)
*C. jamaicensis*	130 (4.9)	21 (4.8)	151 (4.9)
*C. minutissimus*	111 (4.2)	4 (0.9)	115 (3.7)
*C. haranti*	64 (2.2)	0 (0.0)	64 (2.1)
*C. newsteadi*	36 (1.4)	27 (6.1)	63 (2.0)
*C. indistinctus *^*3*^	39 (1.5)	2 (0.5)	37 (1.2)
*C. kurensis* variation ^*4*^	19 (0.7)	13 (3.2)	32 (0.9)
*C. sahariensis*	2 (0.1)	18 (4.1)	20 (0.6)
*C. cataneii*	7 (0.3)	8 (1.8)	15 (0.5)
*C. corsicus*	7 (0.3)	4 (0.9)	11 (0.4)
*C. nubeculosus*	2 (0.1)	8 (1.8)	10 (0.3)
*C. saevus*	7 (0.3)	1 (0.2)	8 (0.3)
*C. begueti*	6 (0.2)	1 (0.2)	7 (0.2)
*C. festivipennis*	7 (0.3)	0 (0.0)	7 (0.2)
*C. longipennis*	5 (0.2)	1 (0.2)	6 (0.2)
*C. shaklawensis*	4 (0.2)	0 (0.0)	4 (0.1)
*C. jumineri* near *bahrainensis *^*5*^	0 (0.0)	3 (0.7)	3 (0.1)
*C. montanus*	2 (0.1)	0 (0.0)	2 (0.1)
*C. haranti* variation ^*6*^	2 (0.1)	0 (0.0)	2 (0.1)
*C. helveticus*	2 (0.1)	0 (0.0)	2 (0.1)
*C. fagineus*	2 (0.1	0 (0.0)	2 (0.1)
*C. gejgelensis*	1 (< 0.1)	0 (0.0)	1 (< 0.1)
Undetermined spp. ^*7*^	5 (0.2)	1 (0.2)	6 (0.2)
Damaged	392 (14.8)	67 (15.2)	459 (14.9)
Total	2644	440	3084

^1,2^ Represent a new species and a new record for Europe, respectively

^3,4,6^ These species show minor *sensilla coeloconica* variations and do not match with original descriptions

^5^ Represent a new record for Spain, but it is not already defined as a valid species

^7^ Five taxa undetermined represent specimens with unique features but due to the low numbers we cannot infer if they represent valid species or are atypical variations or abnormal specimens

### *Culicoides* abundance and species richness

In this study, the most common species was *C. imicola* (17.4%), followed by *C. grandifovea* sp. nov. (14.6%), *C. kurensis* (11.9%), and *C. pseudolangeroni* (11.7%). Collections of the remaining species were much less abundant (Table 2). Based on the frequency of trapping, *C. circumscriptus* was recorded as the most widely distributed species (122 times), followed by *C. pseudolangeroni* (55 times), *C. imicola* (48 times), and *C. grandifovea* sp. nov. (14 times). Due to missing, broken, or damaged parts of their bodies, 14.9% of the specimens could not be identified at species level. Out of the 23 collected *Culicoides* species, eight were plain-winged specimens and 15 were wing patterned species. Out of the 476 sampling sites (approximately 1786 days of trapping effort), 225 times (12.5%) were positive for *Culicoides* species (Fig. 1), including 118 (6.6%) in spring, 65 (3.6%) in summer, and 42 (2.3%) in autumn.

### Flight seasonality of *Culicoides* species

**Fig. 2:**
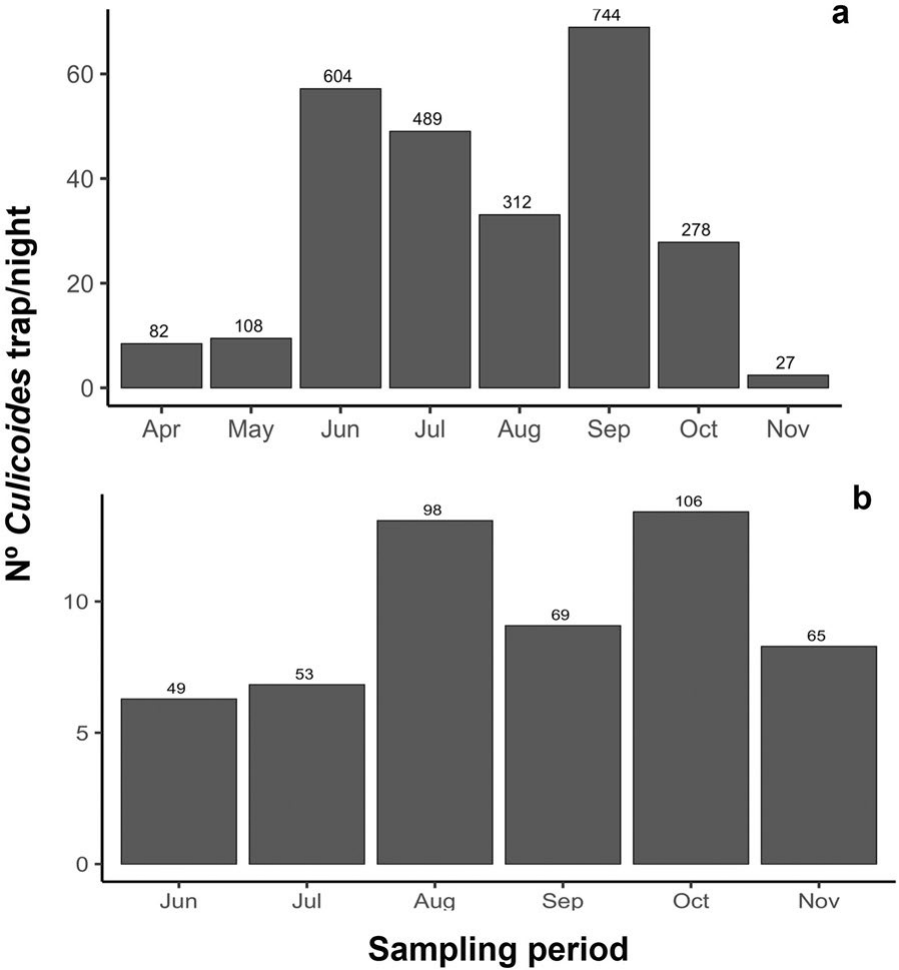
Number of *Culicoides* spp. per trap per night collected in Andalusia (southern Spain) in 476 sampling sites with carbon dioxide baited BG-traps. **a** 450 localities sampled in three occasions between April and November 2023. **b** 26 localities sampled at weekly or 2-weeks intervals between June and November 2023. The number above the bar represents the number of trapped individuals in each period

*Culicoides* spp. (all species gathered) remained active throughout the entire period of sampling (Fig. 2), with a peak in September and decrease in November. Similar numbers of *Culicoides* were captured in May, June, October, and November, while the minimum *Culicoides* numbers were collected in August, coinciding with the hottest month in Andalusia (Fig. 2). *Culicoides imicola* was more commonly collected in September followed by July, but the peak of *C. imicola* captures was more notorious in the second survey, where they peaked in autumn (October and November). *Culicoides kurensis,* the third most common species, followed a similar trend than *C. imicola.* Moreover, *C. grandifovea* sp. nov. showed the opposite pattern than *C. imicola* and *C. kurensis. Culicoides grandifovea* sp. nov. exhibited moderate peaks from May to August, while was absent in autumn (Supplementary Fig. 1).

### New recorded species

**Fig. 3:**
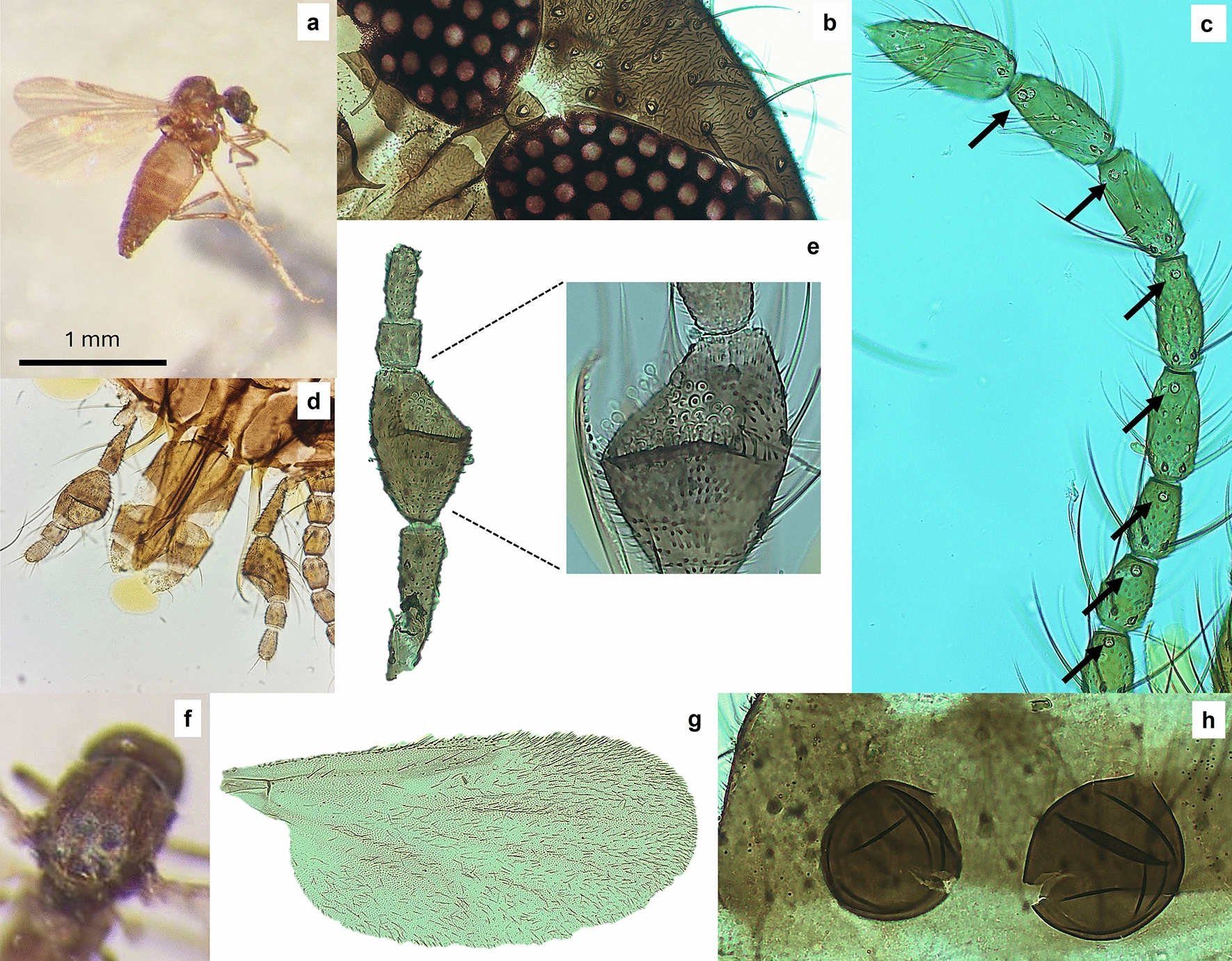
Habitus of *Culicoides grandifovea* sp. nov. **a** General aspect. **b** Interocular space. **c** Antennal *sensilla coeloconica* distribution. **d** Maxillary palpus. **e** Sensory pit in the third palpus segment. **f** *Scutum*. **g** Wing pattern. **h** Spermathecae

* Culicoides grandifovea* sp. nov. González, 2024 (Fig. 3).

ZooBank registration: details of the new species have been submitted to ZooBank. The LSID for the new name *Culicoides grandifovea* is urn: https://zoobank.org/References/12517686-f069-49b4-9c77-46d1dd617cfc

Type material: holotype (*n* = 1 female) from Huelva province (2/08/2023; 37.5230120 -6.5379540)  (Andalusia) collected by a BG-sentinel 2 trap supplemented with dry ice and deposited in the Entomology collection of the Estación Biológica de Doñana (CODE: 2014.060; EBD-CSIC, Sevilla, Spain) (https://www.csis.es) along with  paratypes (n = 3).  Specimens stored in ethanol (70%) (1.5 Eppendorf tubes) and slide-mounted specimens (*n* = 10) also available upon request. https://www.ebd.csic.es/. Coordinates available in Supplementary Table 1.

Habitat: specimens collected in diverse habitats, including mainly Mediterranean scrubs and diverse tree forests such as eucalyptus, pine, olive grove, and Holm oak trees, among others.

Distribution: widely distributed in the provinces of Sevilla, Huelva, and Cádiz (southern Spain) (Fig. 1).

Etymology: based on the large size of the sensory pit of the third palpus segment. Fovea (foʊviə) is Latin for pit. It refers to a pit or depression in the third palpus.

#### Description of female

Size: medium size species (1.3–1.6 mm) (Fig. 3a).

Head: eyes bare, narrowly separated by a distance of approximately three-fourths or one ocular facet (Fig. 3b). Eyes connected by two fine transverse sutures (superior and central) enclosing the interocular setae. Number of *sensilla coeloconica* on flagellomeres III–XIV are 3–4/0–1/1/1/1/1/1/1/1/1/1/1/1/2 (Fig. 3c). Flagellomeres III–XIV with means of length of 49.2/32.5/31.6/32.6/32.9/32.4/31.8/32.2/48.2/48.4/50.7/53.1/67.5 µm. Mean AR of 0.97 (0.95–1.03 µm). Palpi are five-segmented with 238.80 ± 20.86 µm in length (Fig. 3d). The third palpus segment is strongly inflated with a single and wide shallow circular sensory pit (occupying almost the front half) of the palpus and is full of *sensilla* that externally exceed (Fig. 3e). Mean PR of 1.77 (1.65–1.78 µm). Area of sensory pit (third palpus) of 605.20 (68.3) µm^2^. Distance from posterior pharynx to end of hypopharynx: 215.25 (208–227 µm). Mandible with a mean of 11.75 teeth and maxillae with 15.50 teeth. Further details are provided in Supplementary Table 2.

Thorax: scutum dark brown with ornamentation (shown in fresh specimens). Median part of scutum with two broad light bands along the scutum (Fig. 3f). Color varies depending on light incidence. Halteres pale. Unspotted wings with abundant evenly distributed microtrichia, with a little more abundant microtrichia in the apical zone (R_5_ area) (Fig. 3g). Mean length of wings is 1087.3 (1039–1128 µm) and width is 502.1 (468–529 µm). Radial cells (R_1_ and R_2_) are noticeably dark. Inconspicuous marked pale spots on r–m cross vein and second costal area of wings. Legs brown uniform with tarsal segments lighter (slide-mounted specimens). Slender legs, fore (femur = 393–406 µm length and tibia = 388–409 µm), mid (femur = 392–408 µm length and tibia = 379–408 µm), and hind legs (femur = 306–328 µm length and tibia = 334–369 µm), with first tarsomere two times longer than the second one in the three pairs of legs. Tibial comb in fore legs with four major spurs of similar size along with other spines with smaller size. Spines of tarsomeres absent in fore and hind legs and present in tarsomere I–IV in middle legs.

Abdomen: two fully functional spherical spermathecae highly sclerotized (dark brown color). Spermathecae slightly asymmetric (length versus width: 66.5 ± 2.12 µm × 53.0 ± 4.32 µm and 53.0 ± 7.1 µm × 45.6 ± 6.66 µm) with a short unpigmented neck or without neck (Fig. 3h). Third rudimentary spermatheca vestigial. Sclerotized ring and abdominal sclerites absent.

Differential diagnosis and remarks. This species is similar in size and appearance to other dully colored (plain-wing and half-wing species) medium sized species such as *C. kibunensis*, *C. indistinctus*, *C. odiatus*, and other related species. However, the pale spot on both r–m and second costal spot is more marked in these species compared with *C. grandifovea* sp. nov. In addition, the large sensory pit of the third maxillary palpus might be observed under 6–8× magnification for ruling out the previous species. The ornamentation of the thorax is also shared with *C. indistinctus*. Accurate identification requires the elaboration of slide-mounted specimens. Under the compound microscope, *C. grandifovea* sp. nov. is unique since it combines the three following features: (1) distribution of *sensilla coeloconica* from III–XIV (variable in IV) (allowing the exclusion of many other plain-wing species), (2) the third palpal segment is moderately swollen with a large circular shallow sensory pit (in *C. odiatus* and *C. indistinctus* is different) with certain resemblance to the palpi of *C. kurensis*, and (3) spermathecae are slightly asymmetric, highly sclerotized (dark brown color), and spherical without neck. Unspotted wings are overall large and showy. Another less relevant feature is the presence of two sutures joining the eyes. Males were not captured, probably because carbon dioxide traps do not attract them.

### New record of *C. pseudolangeroni*

*Culicoides pseudolangeroni* represents the first record from Europe. Collections of this species (11.8% of the total; 363 females and 1 male from 11 April to 27 October 2023) occurred in the five sampled provinces (Cádiz, Sevilla, Huelva, Málaga, and Córdoba) (Fig. 1). Its body length size is between 0.95–1.15 mm (Supplementary Fig. 2a). Eyes are bare and separated by a distance equal to the diameter of one ommatidial facet (Supplementary Fig. 2b). Thorax is typically brown, unspotted, and covered by visible interspersed setae (Supplementary Fig. 2c). *Scutellum* is usually yellowish or lighter compared with *scutum* (Supplementary Fig. 2d). The functional spermathecae are lightly sclerotized with a short neck and ring present (Supplementary Fig. 2e). Antennae, *sensilla coeloconica* on flagellomeres III–VI and XI–XIV (Supplementary Fig. 2f). Plain wings, with no markings (Supplementary Fig. 2g). Maxillary palpi (third palpus segment) with a single open and shallow sensory pit (Supplementary Fig. 2h). The most characteristic feature of males is the base of the parameters. They bear a highly sclerotized circular-shape structure leading to a pointed protuberance (Supplementary Fig. 2i). Further details are provided in Supplementary Table 2.

### Phylogenetic analysis

**Fig. 4:**
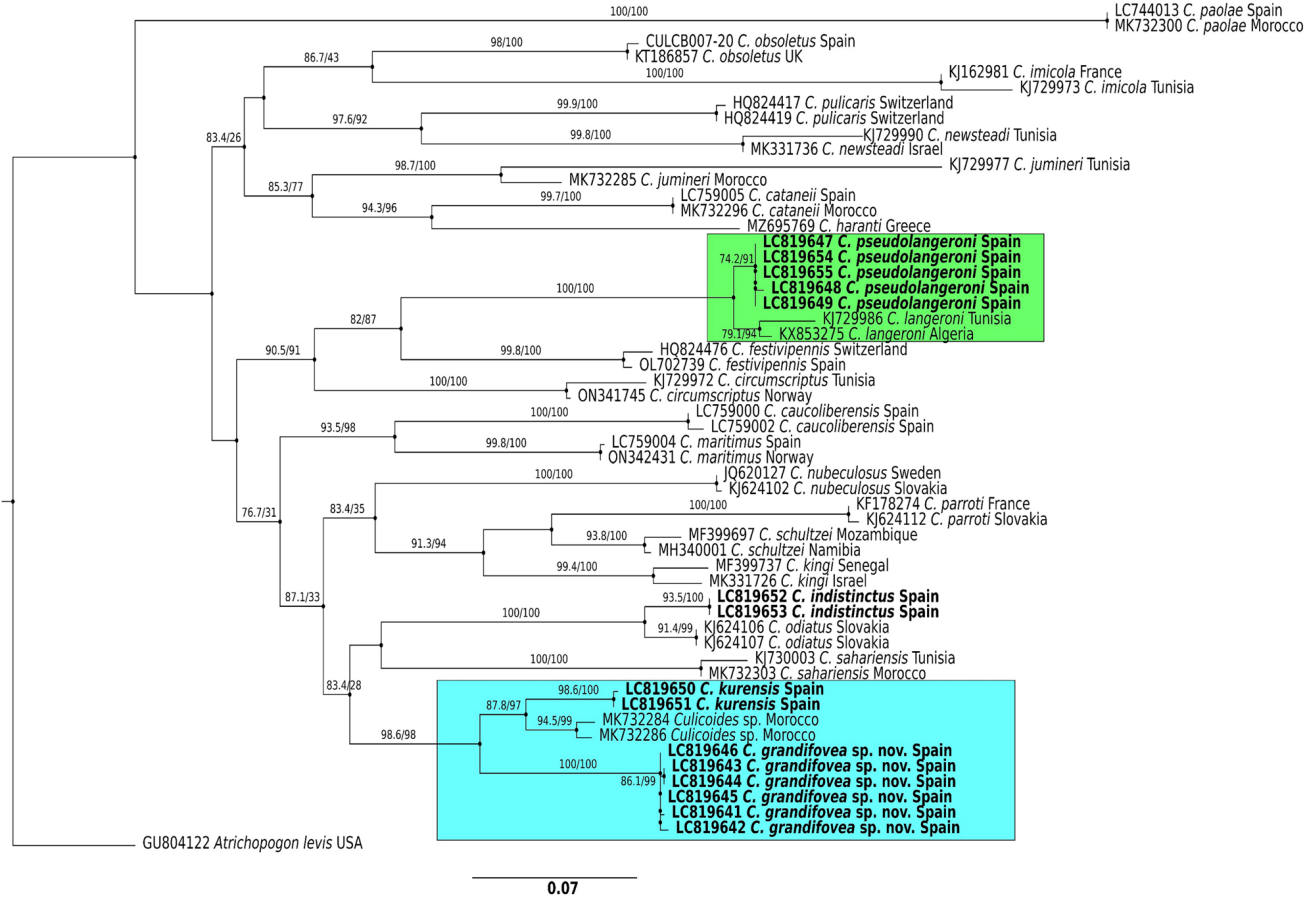
Maximum likelihood (ML) phylogenetic tree based on 55 COI sequences of *Culicoides* species. Topological branch support for the ML analysis (aLRT/bootstrap) is reported over specific branches, with values > 75% defining high stability. The sequences of this study (*n* = 15; four species) are marked in bold

New COI sequences (> 600 pb) have been deposited for *C. grandifovea* sp. nov. (LC819641-46), *C. pseudolangeroni* (LC819647-49, LC819654-55), *C. kurensis* (LC819650-51), and *C. indistinctus* (LC819652-53). Intraspecific *d* values were very low (*d* = 0.002%) among *C. grandifovea* sp. nov. sequences. Similarity of *C. grandifovea* COI sequences with other *Culicoides* sequences were lower than 92.6%. The more similar sequences corresponded to an unknown *Culicoides* sp. (MK732284 and MK732286) captured in Morocco and *C. kurensis*, forming a cluster separate from the rest of the species of the subgenus *Oecacta* (blue box, Fig. 4). Two out of the six specimens analyzed by barcoding showing lack of *sensilla coeloconica* in segment IV resulted to be genetically similar to the other four *C. grandifovea* sp. nov. showing such sensilla.

Similarity of *C. pseudolangeroni* sequences in relation with the closely related species *Culicoides langeroni* (KJ729987) ranged between 98.06% and 97.25%. According to the phylogenetic tree, *C. pseudolangeroni* is a single monophyletic group (green box, Fig. 4) with an intraspecific d value (*d* = 0.001%) for *C. pseudolangeroni*, albeit clustered alongside *C. langeroni*. The genetic distance between *C. pseudolangeroni* and *C. langeroni* COI sequences ranged between 2% and 3% (Supplementary Table 3).

## Discussion

This study provides a comprehensive faunistic catalog of the *Culicoides* species found in southern Spain, an area historically affected by *Culicoides*-borne pathogens [29, 59]. The use of nonstandard sampling methods carried out in diverse environments helped in identifying a high diversity of species, including some previously unknown species in this region. Interestingly, using this approach, two of the three most common species recorded in the area resulted to be a new species for science and a new record for Europe. In addition, many of the remaining collected *Culicoides* species are poorly documented in literature.

Light or carbon dioxide are the most commonly used baits for the collection of *Culicoides* midges [60, 61]. Due to the strong attraction of UV-light sources, *Culicoides* are usually collected in high numbers, especially in livestock farms and natural landscapes where wild ruminants are present [60, 62]. Light traps represent a practical and economical trapping system to determine species presence and abundance in an area [63]. However, light is an artificial attraction stimulus that does not mimic or reflect any response to a host. Several studies have indicated carbon dioxide to be an attractant for a number of blood-feeding insects, including *Culicoides* [64, 65]. However, a limited number of studies have exploited it, as carbon dioxide is relatively expensive, has a short period of operation, and is often considered impractical for routine use in large-scale surveillance programs [22, 63].

Using carbon dioxide traps, we collected a wide range of *Culicoides* species but in lower numbers compared with light traps, which is in line with other studies [61–63, 66]. While we collected a mean of 1.72 *Culicoides* specimens (per trap per day), 10–100 midges (per trap per day) have been collected in studies using light traps in southern Spain [47, 50]. Regarding the species composition, *C. imicola, C. newsteadi*, *C. pulicaris* group, *C. circumscriptus*, and *C. obsoletus* group dominated in previous studies using light traps in farms or in presence of livestock in Andalusia [28, 48, 50, 53], which contrasts with our findings. This difference in species composition might be attributed to different factors including variable grade of attraction to carbon dioxide and the habitat sampled, with species such as those of the Obsoletus and Pulicaris groups usually associated with livestock [20, 67, 68]. In addition, the traps used may affect the ratios, abundances, and species richness of *Culicoides* captured [61, 69–71]. Carbon dioxide-baited traps may be useful to capture host-seeking females for epidemiological studies while UV traps might be used to capture blood-fed females for host identification analysis [61, 65, 66, 72]. In fact, only six specimens with blood were collected in our study (0.2%). We showed that carbon dioxide baited traps were successful for the collection of the main Afrotropical vector *C. imicola*, which is consistent with other studies [64]. However, no collections were made of the widespread species *C. obsoletus*, probably because this species responds poorly to carbon dioxide-baited traps [66, 73–75].

Overall, the *Culicoides* fauna of southern Spain comprises species with different geographical distributions such as Palearctic, Mediterranean Basin, and Afrotropical species [76]. About 35% of the *Culicoides* species collected belonged to the so-called group of “plain-wing species” and/or poorly developed wing pattern species, which are predominant in drier and more open habitats [77]. Interestingly, among the recorded species, we found the new species *C. grandifovea* sp. nov. with a broad distribution in southern Spain and relatively high abundance in the area. The molecular analyses of the barcoding region of this species support a single genetic cluster group, with the closer phylogenetic relations with sequences from unidentified *Culicoides* from Morocco and with *C. kurensis*. *Culicoides grandifovea* sp. nov. displayed a flight activity with a major peak in the hottest and driest month of the year. The fact that this second most abundant species was not recorded previously might indicate its absence in farmland habitats and/or a low attraction to light traps. Also, active trapping conducted in previous years with suction light traps in the region have revealed absence of this species (data not shown). Based on the distribution of *sensilla coeloconica* and the palpus size, a preference of this species to feed on avian blood might be expected [78]. We also recorded *C. pseudolangeroni* for the first time in Europe. This species belongs to the *C. langeroni* species group, together with *C. langeroni*, *Culicoides judae*, and *Culicoides molotovae* [79], which is in line with the phylogenetic tree results. *Culicoides pseudolangeroni* has been previously found in deserts of Central Asia and North Africa [14, 80–82]. In addition, at least three individuals of *C. jumineri* near *bahrainensis* were captured in this study. The taxonomy of the Jumineri species group is not yet studied and the taxonomic status of the species remains unclear. Unfortunately, we were unable to recover DNA from these specimens, as they were mounted in slides. A molecular analysis comparing the nucleotide sequences of genes such as COI and/or ITS2 is needed to resolve the issue between *C. jumineri* s.s. and *C. bahrainensis* s.s., the latter distributed in Saudi Arabia [83]. In addition, three *Culicoides* variations (*C. haranti* variation, *C. kurensis* variation and *C. indistinctus*) were recorded. These kinds of variations are frequently recorded in literature [14, 84]. Also, five unknown *Culicoides* taxa were recorded in low numbers (≤ 2 specimens each one). These species possess *sensilla coeloconica* variations in antennal flagellomeres and/or other features (atypical pit shape); however, more specimens are necessary to determine if they represent valid species. This material evidences the complexity of the taxonomy of the *Culicoides* genus.

Regarding BTV, AHSV, and EHD virus disease vectors, *C. obsoletus* and *C. pulicaris* group species were less predominant and geographically temporally distributed than *C. imicola*, but they are frequently reported in southern Spain [45, 50, 53]. It is interesting to note the absence of members of *C. pulicaris/C. lupicaris* and *C. obsoletus* groups except two specimens of *C. montanus*. This can be due to different nonexclusive causes. First, latter species are usually associated with farm environments, and second, it might be possible that *C. montanus* can be overlooked with *C. obsoletus* and *Culicoides scoticus* species. However, they can be easily separated from the other members of the *Obsoletus* group under the stereo microscope by observing the single deep pit of the palpi. Similar reasons might explain that some species were not recorded in the past by light suction traps, being some of them particularly abundant in our study (*C. kurensis*, *C. grandifovea* sp. nov., and *C. pseudolangeroni*). Ornithophilic species such as *C. circumscriptus* and *C. jamaicensis* (previously named *C. paolae*) commonly captured in the region by different trapping methods [38], have resulted also abundant in our carbon dioxide baited traps.

Finally, our results provide information on the seasonal activity of *Culicoides* species in the area. Although variable between species, the flight activity of *Culicoides* spp. (including *C. imicola*) showed a major peak at the end of spring and another one in September/October. These results agree with previous studies, where *C. imicola* peaked between August and November with a remarkable variation depending on sites [28, 45, 48, 53]. Abiotic and biotic parameters including climatic variables such as precipitation and temperature may determine the abundance of *Culicoides* species in the area [28, 48, 50, 52, 85]. This may be especially relevant due to the low rainfall (totally absent in mid-summer) and extremely high temperatures (July–August) in the study area which may impact the development and/or flight activity of most *Culicoides* species.

## Conclusions

Through a comprehensive literature review alongside extensive active trapping, we have expanded the known *Culicoides* fauna in Spain to 88 valid species, specifically to 53 in southern Spain. Our findings underscore the importance of complementing traditional UV-light traps with alternative trapping methods such as carbon dioxide-baited traps to comprehensively assess *Culicoides* abundance and distribution. This system allowed us to identify a previously undescribed species of *Culicoides* despite its widespread distribution and abundance in the area. Future studies should determine the role of these new or poorly documented *Culicoides* species in the transmission of pathogens of interest in animal and public health.

### Supplementary Information

Additional file 1.

## Data Availability

Data is provided within the manuscript or supplementary information files.

## Abbreviations

UV: Ultraviolet
AHSV: African horse sickness virus
BTV: Bluetongue virus
SV: Schmallenberg virus
EHD: Epizootic hemorrhagic disease
OVI: Onderstepoort veterinary institute
COI: Cytochrome c oxidase subunit I
AR: Antennal ratio
PR: Palpal ratio
LISD: Life science identifiers
