# Supplementary material for: Sampling of Culicoides with nontraditional methods provides unusual species composition and new records for southern Spain
Source: Parasit Vectors. 2024 Aug 12;17:338. doi: 10.1186/s13071-024-06414-2 (PMC11318182; doi:10.1186/s13071-024-06414-2)
Supplement: Supplementary file 1 — Additional file 1. [file 13071_2024_6414_MOESM1_ESM.docx]

**Supplementary information**

| **Month** | **Coordinates** | **Nº specimens** | **Habitat** |
| --- | --- | --- | --- |
| May | 36.297045, -5.867136 | 2 | Olive grove |
| May | 37.543276, -7.046505 | 2 | Scrubs |
| June | 37.635514, -6.627346 | 15 | Riparian vegetation |
| June | 37.344600, -6.639984 | 110 | Scrubs |
| June | 37.767019, -7.028623 | 37 | Woodland |
| July | 37.3165432, -7.3548337 | 6 | Scrubs |
| July | 37.52843, -7.272591 | 1 | Scrubs |
| July | 37.548127, -6.051914 | 2 | Urban trees |
| July | 37.519279, -5.859416 | 1 | Eucalyptus forest |
| July | 37.3664662, -4.9868029 | 23 | Eucalyptus forest |
| August | 37.5230120, -6.5379540 | 230 | Holm oak |
| August | 37.914752, -7.194055 | 18 | Scrubs |
| August | 37.160235, -6.807134 | 1 | Mixed pine forest |
| October | 36.308543, -5.724029 | 1 | Mediterranean forest |

**Table 1.** Details of *C. grandifovea* sp. nov. collections with BG traps baited with carbon dioxide in Andalusia (southern Spain) in 2023.

**Table 2.** Morphometric parameters of *C. grandifovea* sp. nov. and *C. pseudolangeroni* measured in microns from 10 individuals of each species*.* SD = Standard Deviation. Length and width measures are expressed in microns.

| **Parameters** | ***C. pseudolangeroni*** | ***C. grandifovea* sp. nov** |
| --- | --- | --- |
| Distribution of *sensilla coeloconica* | 3-6, 11-14 | 3-14 |
| Nº of *sensilla* *coeloconica* per flagellomere | (3-4),1,1,1,1,1,1, (1-2) | (3-4),(0-1),1,(0-1),1,1,1,(1-2) |
| Wing length ± SD | 875.9 ± 50.41 | 1087.3 ± 77.10 |
| Wing width ± SD | 424.6 ± 13.1 | 502.1 ± 34.79 |
| Length of spermathecae ± SD | 39.2 ± 7.59 x 42.1± 4.34 | 66.5 ± 2.12 x 55.0 ± 4.32 |
| Width of spermathecae ± SD | 34.0 ± 4.54 x 37.3 ± 5.48 | 53.0 ± 7.1 x 45.6 ± 6.66 |
| Nº of mandibular teeth, mean (min-max) | 14.2 (14-15) | 11.75 (9-14) |
| Nº of maxillary teeth, mean (min-max) | 15.6 (15-16) | 15.5 (15-17) |
| Length palpus (1-5 segments) ± SD | 210.8 ± 20.10 | 238.8 ± 20.86 |
| Length palpus (3^rd^ segment) ± SD | 63.8 ± 7.25 | 69.5 ± 7.10 |
| Width palpus (3^rd^ segment) ± SD | 32.8 ± 3.25 | 45.3 ± 3.80 |
| Area of the sensory pit, mean ± SD | NA | 605.2 ± 68.31 |

**Table 3.** Estimation of the evolutionary distance between different *Culicoides* species for the COI gene. Standard error estimate(s) are shown above the diagonal and were obtained by a bootstrap procedure (1000 replicates). This analysis involved 21 nucleotide sequences. All ambiguous positions were removed for each sequence pair (pairwise deletion option). There was a total of 766 positions in the final dataset. Evolutionary analyses were conducted in MEGA11.

| ***Culicoides* species** | **1** | **2** | **3** | **4** | **5** | **6** | **7** | **8** | **9** | **10** | **11** | **12** | **13** | **14** | **15** | **16** | **17** | **18** | **19** | **20** | **21** |
| --- | --- | --- | --- | --- | --- | --- | --- | --- | --- | --- | --- | --- | --- | --- | --- | --- | --- | --- | --- | --- | --- |
| 1_C4_*C. grandifovea* sp. nov. Spain |  | 0.00224 | 0.00239 | 0.00237 | 0.00163 | 0.00163 | 0.01317 | 0.01300 | 0.02365 | 0.01711 | 0.02496 | 0.02020 | 0.01930 | 0.01968 | 0.01930 | 0.02016 | 0.01930 | 0.01826 | 0.01821 | 0.02037 | 0.02233 |
| 2_C5_ *C. grandifovea* sp. nov. Spain | 0.00321 |  | 0.00224 | 0.00227 | 0.00154 | 0.00218 | 0.01308 | 0.01292 | 0.02362 | 0.01693 | 0.02434 | 0.01952 | 0.01864 | 0.01892 | 0.01855 | 0.01970 | 0.01864 | 0.01811 | 0.01774 | 0.01993 | 0.02143 |
| 3_C12_ *C. grandifovea* sp. nov. Spain | 0.00324 | 0.00315 |  | 0.00000 | 0.00162 | 0.00161 | 0.01322 | 0.01326 | 0.02379 | 0.01680 | 0.02455 | 0.01986 | 0.01907 | 0.01955 | 0.01915 | 0.01962 | 0.01929 | 0.01853 | 0.01825 | 0.01976 | 0.02157 |
| 4_C13_ *C. grandifovea* sp. nov. Spain | 0.00323 | 0.00319 | 0.00000 |  | 0.00163 | 0.00163 | 0.01322 | 0.01320 | 0.02360 | 0.01728 | 0.02488 | 0.02042 | 0.01963 | 0.01995 | 0.01963 | 0.02037 | 0.01963 | 0.01844 | 0.01816 | 0.02038 | 0.02236 |
| 5_C14_C *C. grandifovea* sp. nov. Spain | 0.00160 | 0.00156 | 0.00159 | 0.00159 |  | 0.00000 | 0.01308 | 0.01291 | 0.02347 | 0.01691 | 0.02469 | 0.01983 | 0.01896 | 0.01928 | 0.01896 | 0.01992 | 0.01896 | 0.01818 | 0.01793 | 0.02011 | 0.02182 |
| 6_C15_ *C. grandifovea* sp. nov. Spain | 0.00160 | 0.00307 | 0.00158 | 0.00159 | 0.00000 |  | 0.01308 | 0.01290 | 0.02350 | 0.01687 | 0.02452 | 0.01949 | 0.01867 | 0.01881 | 0.01859 | 0.01974 | 0.01867 | 0.01811 | 0.01774 | 0.01996 | 0.02146 |
| 7_C7_*C. kurensis* Spain | 0.09551 | 0.09478 | 0.09658 | 0.09658 | 0.09478 | 0.09478 |  | 0.00160 | 0.02248 | 0.01717 | 0.02415 | 0.01852 | 0.01727 | 0.01762 | 0.01727 | 0.01762 | 0.01727 | 0.01839 | 0.01822 | 0.01964 | 0.02183 |
| 8_C8_ *C. kurensis* Spain | 0.09420 | 0.09390 | 0.09658 | 0.09609 | 0.09317 | 0.09383 | 0.00162 |  | 0.02210 | 0.01697 | 0.02447 | 0.01840 | 0.01705 | 0.01744 | 0.01697 | 0.01786 | 0.01705 | 0.01775 | 0.01736 | 0.01971 | 0.02148 |
| 9_JQ620127_*C. nubeculosus* Sweden | 0.18048 | 0.18444 | 0.18080 | 0.17940 | 0.17911 | 0.18123 | 0.17245 | 0.17031 |  | 0.02323 | 0.02459 | 0.02546 | 0.02498 | 0.02493 | 0.02487 | 0.02649 | 0.02498 | 0.02535 | 0.02512 | 0.02802 | 0.02918 |
| 10_LC759005_*C. cataneii* Spain | 0.16025 | 0.16381 | 0.15994 | 0.16533 | 0.16164 | 0.16221 | 0.15777 | 0.15888 | 0.18429 |  | 0.02573 | 0.02090 | 0.02018 | 0.02062 | 0.02006 | 0.02069 | 0.02036 | 0.02001 | 0.01952 | 0.02083 | 0.02297 |
| 11_C9_*C. indistinctus* Spain | 0.16571 | 0.16421 | 0.16991 | 0.16897 | 0.16480 | 0.16421 | 0.16044 | 0.15461 | 0.21348 | 0.19260 |  | 0.02688 | 0.02611 | 0.02614 | 0.02593 | 0.02690 | 0.02611 | 0.02656 | 0.02625 | 0.02869 | 0.02946 |
| 12_C10_ *C. indistinctus* Spain | 0.16814 | 0.16372 | 0.17043 | 0.16951 | 0.16546 | 0.16372 | 0.16377 | 0.15518 | 0.21129 | 0.19090 | 0.00000 |  | 0.00636 | 0.00693 | 0.00635 | 0.00576 | 0.00641 | 0.02136 | 0.02099 | 0.02003 | 0.02139 |
| 13_C1_*C. pseudolangeroni* Spain | 0.18870 | 0.18272 | 0.18696 | 0.19172 | 0.18531 | 0.18304 | 0.17130 | 0.16937 | 0.21293 | 0.19664 | 0.20098 | 0.20036 |  | 0.00219 | 0.00000 | 0.00000 | 0.00000 | 0.02029 | 0.02001 | 0.01898 | 0.02068 |
| 14_C3_ *C. pseudolangeroni* Spain | 0.18870 | 0.18177 | 0.18762 | 0.19172 | 0.18531 | 0.18208 | 0.17130 | 0.16847 | 0.21129 | 0.19560 | 0.20098 | 0.19927 | 0.00000 |  | 0.00218 | 0.00233 | 0.00220 | 0.02062 | 0.02023 | 0.01921 | 0.02129 |
| 15_C17_ *C. pseudolangeroni* Spain | 0.18870 | 0.18272 | 0.18897 | 0.19172 | 0.18531 | 0.18304 | 0.17130 | 0.16937 | 0.21293 | 0.19804 | 0.20098 | 0.20036 | 0.00000 | 0.00000 |  | 0.00000 | 0.00000 | 0.02029 | 0.01992 | 0.01898 | 0.02088 |
| 16_C2_ *C. pseudolangeroni* Spain | 0.19294 | 0.18708 | 0.19244 | 0.19596 | 0.18943 | 0.18547 | 0.17543 | 0.17414 | 0.21323 | 0.20187 | 0.20546 | 0.20358 | 0.00308 | 0.00307 | 0.00309 |  | 0.00000 | 0.02129 | 0.02094 | 0.01927 | 0.02155 |
| 17_C16_ *C. pseudolangeroni* Spain | 0.19167 | 0.18731 | 0.18729 | 0.19336 | 0.18908 | 0.18766 | 0.17065 | 0.17312 | 0.21959 | 0.19544 | 0.20689 | 0.20644 | 0.00000 | 0.00000 | 0.00000 | 0.00327 |  | 0.02029 | 0.02001 | 0.01899 | 0.02104 |
| 18_HQ824417_*C. pulicaris* Switzerland | 0.19386 | 0.19435 | 0.18800 | 0.19272 | 0.19227 | 0.19132 | 0.18372 | 0.19310 | 0.20509 | 0.21146 | 0.23208 | 0.22952 | 0.22806 | 0.22617 | 0.22806 | 0.22826 | 0.22976 |  | 0.00000 | 0.02163 | 0.02398 |
| 19_KJ729987_*C. langeroni* Tunisia | 0.20579 | 0.20139 | 0.20141 | 0.20660 | 0.20187 | 0.19967 | 0.19008 | 0.19045 | 0.22487 | 0.20945 | 0.21893 | 0.21877 | 0.02501 | 0.02497 | 0.02524 | 0.02979 | 0.01981 | 0.24059 |  | 0.02186 | 0.02389 |
| 20_KJ162981_*C. imicola* France | 0.20113 | 0.19870 | 0.19732 | 0.20376 | 0.20049 | 0.19906 | 0.19171 | 0.19376 | 0.25647 | 0.22429 | 0.22375 | 0.23350 | 0.19330 | 0.19330 | 0.19268 | 0.19609 | 0.19172 | 0.25425 | 0.20976 |  | 0.00673 |
| 21_KJ729973_ *C. imicola* Tunisia | 0.23822 | 0.23394 | 0.23265 | 0.24105 | 0.23540 | 0.23247 | 0.22885 | 0.22807 | 0.27690 | 0.25859 | 0.26095 | 0.26795 | 0.22263 | 0.22501 | 0.22582 | 0.22943 | 0.22745 | 0.27344 | 0.23668 | 0.02703 |  |

**
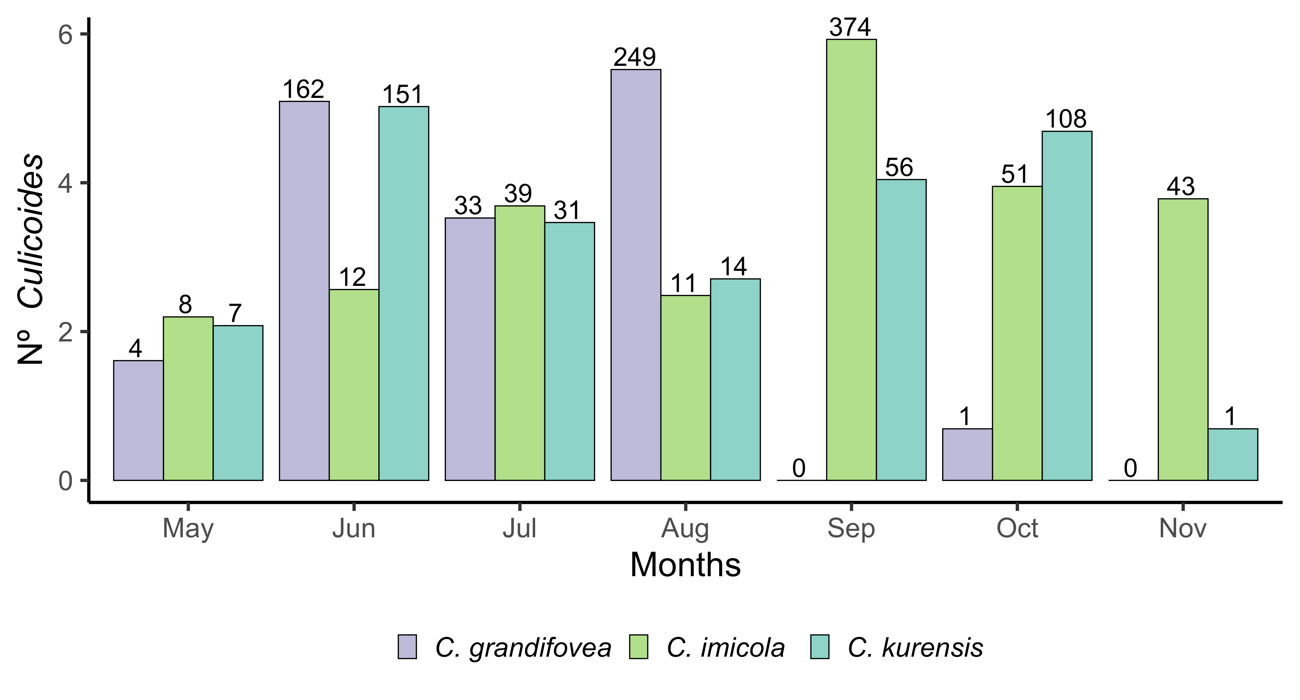
**

**Figure 1.** Number of individuals per trap night for the three most abundant *Culicoides* species collected at 476 sampling sites between April and November 2023 with carbon dioxide baited traps. The number above the bar represents the total number of collected individuals.

**
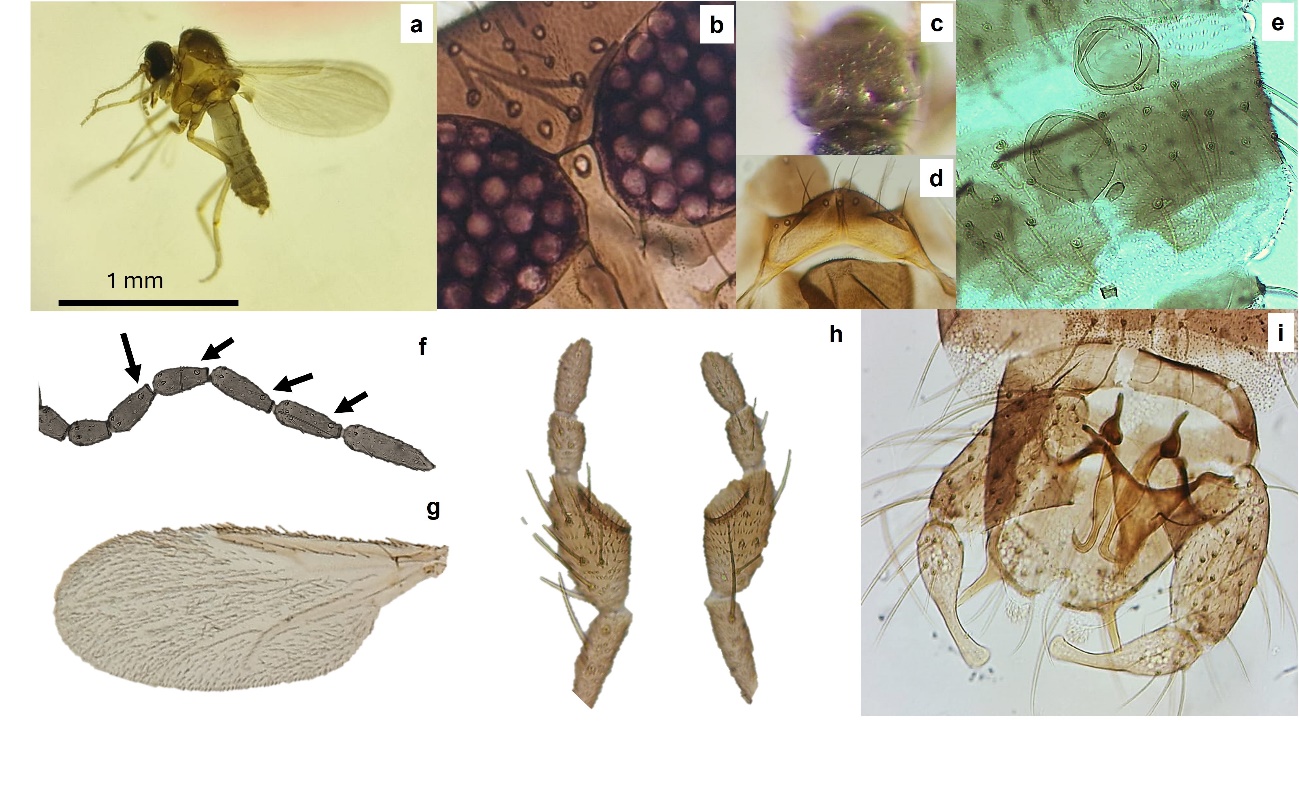
 Figure 2.** Habitus of *C. pseudolangeroni.* **a** General aspect. **b** Interocular space. **c** *Scutum*. **d** *Scutellum*. **e** Spermathecae. **f** Antennal *sensilla coeloconica* distribution. **g** Wing pattern. **h** Maxillary palpus. **i** Male genitalia.
